# Unraveling stroke gait deviations with movement analytics, more than meets the eye: a case control study

**DOI:** 10.3389/fnins.2024.1425183

**Published:** 2024-07-22

**Authors:** Jing Wen Pan, Ananda Sidarta, Tsung-Lin Wu, Wai Hang Patrick Kwong, Poo Lee Ong, Matthew Rong Jie Tay, Min Wee Phua, Wei Binh Chong, Wei Tech Ang, Karen Sui Geok Chua

**Affiliations:** ^1^Rehabilitation Research Institute of Singapore, Nanyang Technological University, Singapore, Singapore; ^2^Department of Sports Science and Physical Education, Chinese University of Hong Kong, Hong Kong, Hong Kong SAR, China; ^3^Department of Rehabilitation Sciences, Hong Kong Polytechnic University, Kowloon, Hong Kong SAR, China; ^4^Institute of Rehabilitation Excellence (IREx), Tan Tock Seng Hospital Rehabilitation Centre, Singapore, Singapore; ^5^Lee Kong Chian School of Medicine, Nanyang Technological University, Singapore, Singapore; ^6^School of Mechanical and Aerospace Engineering, Nanyang Technological University, Singapore, Singapore

**Keywords:** biomechanics, gait analysis, Statistical Parametric Mapping, mobility, kinematic, kinetic, hemiplegia

## Abstract

**Background:**

This study aimed to identify and quantify the kinematic and kinetic gait deviations in post-stroke hemiplegic patients with matched healthy controls using Statistical Parametric Mapping (SPM).

**Methods:**

Fifteen chronic stroke patients [4 females, 11 males; age 53.7 (standard deviation 12.2) years; body mass 65.4 (10.4) kg; standing height 168.5 (9.6) cm] and 15 matched healthy controls [4 females, 11 males; age 52.9 (11.7) years; body weight 66.5 (10.7) years; standing height 168.3 (8.8) cm] were recruited. In a 10-m walking task, joint angles, ground reaction forces (GRF), and joint moments were collected, analyzed, and compared using SPM for an entire gait cycle.

**Results:**

Generally, when comparing the stroke patients’ affected (hemiplegic) and less-affected (contralateral) limbs with the control group, SPM identified significant differences in the late stance phase and early swing phase in the joint angles and moments in bilateral limbs (all *p* < 0.005). In addition, the vertical and anteroposterior components of GRF were significantly different in various periods of the stance phase (all *p* < 0.005), while the mediolateral component showed no differences between the two groups.

**Conclusion:**

SPM was able to detect abnormal gait patterns in both the affected and less-affected limbs of stroke patients with significant differences when compared with matched controls. The findings draw attention to significant quantifiable gait deviations in the less-affected post-stroke limb with the potential impact to inform gait retraining strategies for clinicians and physiotherapists.

## 1 Introduction

Gait impairments affect more than 70% of stroke survivors, who usually exhibit hemiparetic patterns of weakness (Lawrence et al., 2001). A stroke survivor’s ability to independently ambulate a distance of 10 m is indicative of lower limb function and overall motor performance (Kwakkel et al., 2017), and walking speed is often used to evaluate gait performance (Kollen et al., 2006). Furthermore, functional assessments have been applied clinically to evaluate gait performance, lower extremity joint strength, and muscle force (Goldie et al., 1996; Kollen et al., 2006; Smith et al., 2017; Selves et al., 2020). To better characterize post-stroke hemiplegic gait, biomechanical measurements have been extensively conducted. Generally, stroke survivors had a slower walking speed (Tamaya et al., 2020) compared with the controls. In addition, joint range of motion (ROM) or maximum joint angles of the affected limb were smaller in multiple periods in a gait cycle (Tamaya et al., 2020; Nesi et al., 2023). While the existing literature focuses on the affected limb displaying abnormal movement patterns, specific descriptions of the kinematics or kinetics of the contralateral (less-affected) limb are sparse. Given the possible maladaptation of the less-affected limb, precise measurements may provide valuable insights towards understanding the gait patterns (Patterson et al., 2008).

Recent research has highlighted the significance of correctly measuring the nature of impairment and disability in heterogenous stroke populations, intending to prescribe individualized and effective treatments (French et al., 2022). Recent technologies, such as motion capture systems, can systematically examine gait deviations and track rehabilitation outcomes objectively and accurately. While the abovementioned studies have examined the discrete (zero-dimensional, 0D) variables in stroke survivors using instrumented gait analysis systems or motion capture devices (Teixeira-Salmela et al., 2001; Bijleveld-Uitman et al., 2013; Tamaya et al., 2020; Nesi et al., 2023), information about the time-history of these biomechanical variables in a full gait cycle is unclear.

Statistical Parametric Mapping (SPM), as a statistical analysis tool, is able to detect differences in one-dimensional (1D) datasets (e.g., time-varying waveforms for forces, joint angles, joint moments, and electromyography amplitudes) between two or more conditions/groups (Pataky, 2010, 2012). This method has been applied in gait analysis for able-bodied and athletic populations (Mei et al., 2019; Gao et al., 2020), however, data pertaining to stroke gait are sparse. A recent study examined the gait variables using SPM in hemiplegic gait, and observed greater thorax flexion/extension angle during stance phase and greater thorax internal/external rotation angle during the terminal stance phase in the stroke group than the control group (Tamaya et al., 2020).

In gait analysis, a gait cycle has been usually treated as an entire phase from a heel strike (initial contact) of one foot to the next of the same foot, labeled temporally as 0–100%. In the literature, some found significant differences and interpreted the results into certain periods, e.g., ‘during the pre-swing and initial swing phases (55.2–66.5%)’ (Fernández-Vázquez et al., 2023) and ‘terminal-stance phase (31–50%)’ (Park and Yoon, 2021). They may have accepted the fixed cut-off value of 60% to split an entire gait cycle (stance phase: 0–60%, swing phase: 60–100%). While this is valid for a normal population, it can be problematic for stroke survivors, who usually display individual differences in gait impairments, including prolonged stance phase and a higher ratio of stance-to-swing duration (Olney and Richards, 1996). Hence, splitting an entire gait cycle is warranted in SPM analysis (Tamaya et al., 2020), which may provide more focused information regarding abnormal gait patterns. This present study, therefore, aimed to apply SPM to compare the biomechanical variables of both the affected and less-affected limbs in the stance and swing phases of a gait cycle during a 10-m walking task between the stroke and control groups. It was hypothesized that stroke patients’ both limbs would exhibit different biomechanics compared with the control group.

## 2 Method

### 2.1 Study design and setting

This was a cross-sectional, case control study, comparing the gait patterns of a group of chronic stroke patients with an equal number of matched healthy controls (trial registered with www.clinicaltrials.gov, NCT04169594). The latter comprised retrospective data from an Asian-centric movement database of activities of daily living (ADLs) (Liang et al., 2020). The measurements for both the stroke group and control group (the database) were conducted in the same gait laboratory. Stroke participants were referred from an ambulatory rehabilitation clinic of a public rehabilitation hospital.

### 2.2 Participants

All methods of this study were performed in accordance with the Declaration of Helsinki and the ethical approvals were granted by the National Healthcare Group Domain Specific Review Board, Singapore (DSRB reference number: NHG DSRB 2019/00879). The study was registered with www.clinicaltrials.gov, NCT04169594. All participants provided written informed consent. All stroke participants had a history of stroke with a duration exceeding 6 months, and their minimal ambulatory status was indicated by a Functional Ambulation Category (FAC) score (Holden et al., 1984) greater than 4. The detailed inclusion/exclusion criteria can be found in the Supplementary material. This study recruited 15 stroke patients with 1 data point (Table 1, stroke group). The recruitment flowchart (Supplementary Figure S1) and individual demographic and clinical characteristics for the stroke group (Supplementary Table S1). For each stroke patient, one healthy participant, matched by age, gender, height, body mass, and ethnicity, was selected for analysis (Table 1, control group). The matching method was conducted based on the weighted nearest neighbors-based algorithm (Szekér and Vathy-Fogarassy, 2020).

**Table 1:** Participants’ physical characteristics and demographic information (*n* = 30).

	Stroke	Control	*p* value
n	Females (*n* = 4)	Females (*n* = 4)	--
	Males (*n* = 11)	Males (*n* = 11)	--
Ethnicity	Chinese (*n* = 12)	Chinese (*n* = 14)	--
	Indian (*n* = 1)	Indian (*n* = 1)	--
	Others^**^ (*n* = 2)	Others (*n* = 0)	--
Age (years)	53.7 (12.2)	52.9 (11.7)	0.844
Body mass (kg)	65.4 (10.4)	66.5 (10.7)	0.764
Standing height (cm)	168.5 (9.6)	168.3 (8.8)	0.936
Body mass index (kg/m^2^)	22.9 (2.3)	23.4 (2.1)	0.562
Stroke side^*^	Left (*n* = 5)	--	--
	Right (*n* = 10)	--	--
Stroke diagnosis	Hemorrhage (*n* = 7)	--	--
	Infarct (*n* = 8)	--	--

^*^Stroke side refers to the body side affected (paretic side). Data are expressed as mean (standard deviation). Differences between the stroke group and control group were compared using independent *t*-tests. ^**^Others: Myanmarese (*n* = 1) and Nepalese (*n* = 1).

### 2.3 Data acquisition

All 15 stroke patients and 15 control participants were instructed to perform a 10-m walk (Liang et al., 2020) at a comfortable speed, whereby the data of the 15 controls were retrospectively collected. Only the trials with sufficient marker trajectories and the entire foot planting on the force platforms were used for analysis. The mean (standard deviation) speeds were 0.90 (0.24) m/s for the stroke group and 1.62 (0.27) m/s for the control group, respectively. A certified physiotherapist was present with all 15 stroke participants throughout all trials but did not need to assist them. Participants were able to walk independently with their own footwear and lower limb orthotics (e.g., ankle foot orthoses) as needed.

To facilitate data collection, a 3D motion capture system with 16 two-megapixel Miqus M3 cameras (200 Hz, Qualisys AB, Göteborg, Sweden) was employed with a modified Calibrated Anatomical System Technique (CAST) marker set (Liang et al., 2020). There were 30 retro-reflective markers (12.5 mm) placed on the anatomical landmarks on the trunk, hip, and both lower limbs, and 16 markers as the tracking markers (4 on each of the 4 marker clusters) on the thighs and shanks bilaterally (Liang et al., 2020). Two adjacent force platforms (2000 Hz, type 9260AA6, Kistler Instruments AG, Winterthur, Switzerland) were synchronized with the Qualisys system to record ground reaction forces (GRF). Kinematic data were identified for both lower limbs for the stroke group and control group, and kinematic data were obtained for the torso.

### 2.4 Data analyses

Raw marker trajectories and GRF data were low-pass filtered using a fourth-order Butterworth filter at the cut-off frequencies of 15 Hz (Alhossary et al., 2022) and 50 Hz, respectively on Visual3D (v2021.04.1, C-Motion Inc., Germantown, MD, United States). For each foot, the first initial contact and foot-off events used for analysis were determined according to the vertical GRF threshold of 20 N (Park and Yoon, 2021). As the subsequent initial contact occurred when the foot stepped out of the force platforms, it was determined based on the heel marker trajectories (Tamaya et al., 2020). Two sub-phases in a gait cycle, namely stance and swing phases, were subsequently obtained. Biomechanical variables, including GRF, joint angles, and joint moments, were then calculated using the data obtained through the 3D motion capture system and force platforms, and exported into three planes, respectively, i.e., sagittal (e.g., flexion/extension), frontal (e.g., adduction/abduction), and transverse planes (e.g., internal/external rotation). Joint angles were calculated as Euler angles in accordance with a Cardan rotation sequence of “X-Y-Z” (Cole et al., 1993; Wu et al., 2002). Torso angle was computed as the thorax segment with respect to the pelvis. Due to the stroke impacting one body side, the torso movements are usually asymmetric among stroke patients during ambulation (Van Criekinge et al., 2017), and hence, the torso angle was only analyzed for the gait cycle of the affected limb (Yen et al., 2019). For example, if a stroke patient had the left limb paretic, the torso angle was obtained in the stance and swing phases of the left leg. GRF data were normalized to the individual body weight (N/N), and joint moments were normalized to the individual body mass (Nm/kg) (Park and Yoon, 2021).

### 2.5 Statistical analyses

All kinematic and kinetic data were time normalized to 101 data points for each of the stance and swing phases. Subsequently, the time-normalized variables for each participant were averaged across repetitions to obtain a subject-level dataset. These variables were compared between the stroke and control groups using the t-test function of SPM on Python. Matched right and left limbs of the stroke patients and control participants were selected for analysis. For example, if a stroke participant’s affected limb was the left limb, the left limb of the matched participant was selected for comparison, and vice versa. All statistical tests were set at *α* = 0.05.

## 3 Results

**Figure 1:**
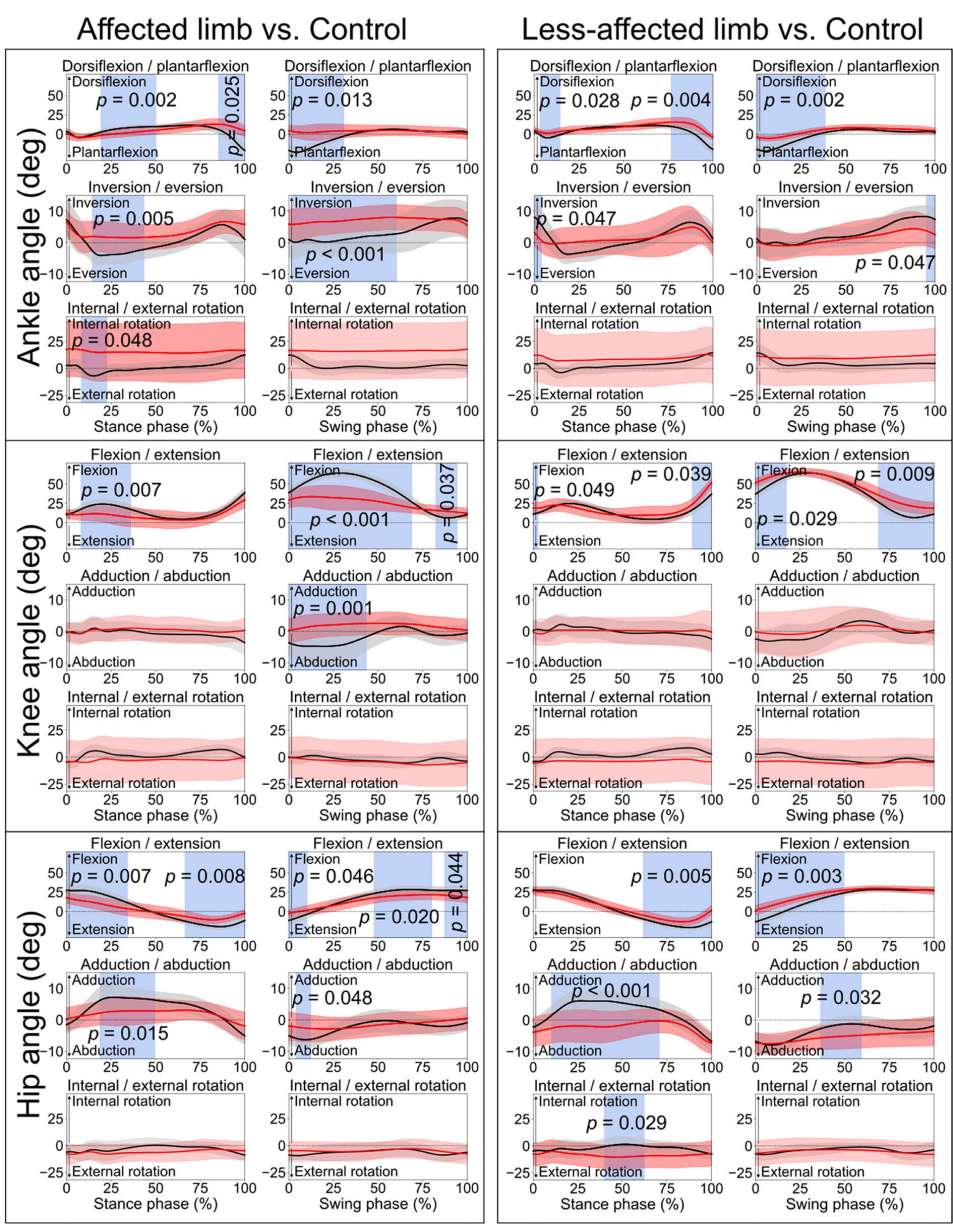
Lower limb joint angles. Comparisons between the stroke group (red lines) and control group (black lines) in the stance phase and swing phase of a gait cycle. Blue shades indicate the time clusters with significant differences between the two groups (*p <* 0.05).

**Figure 2:**
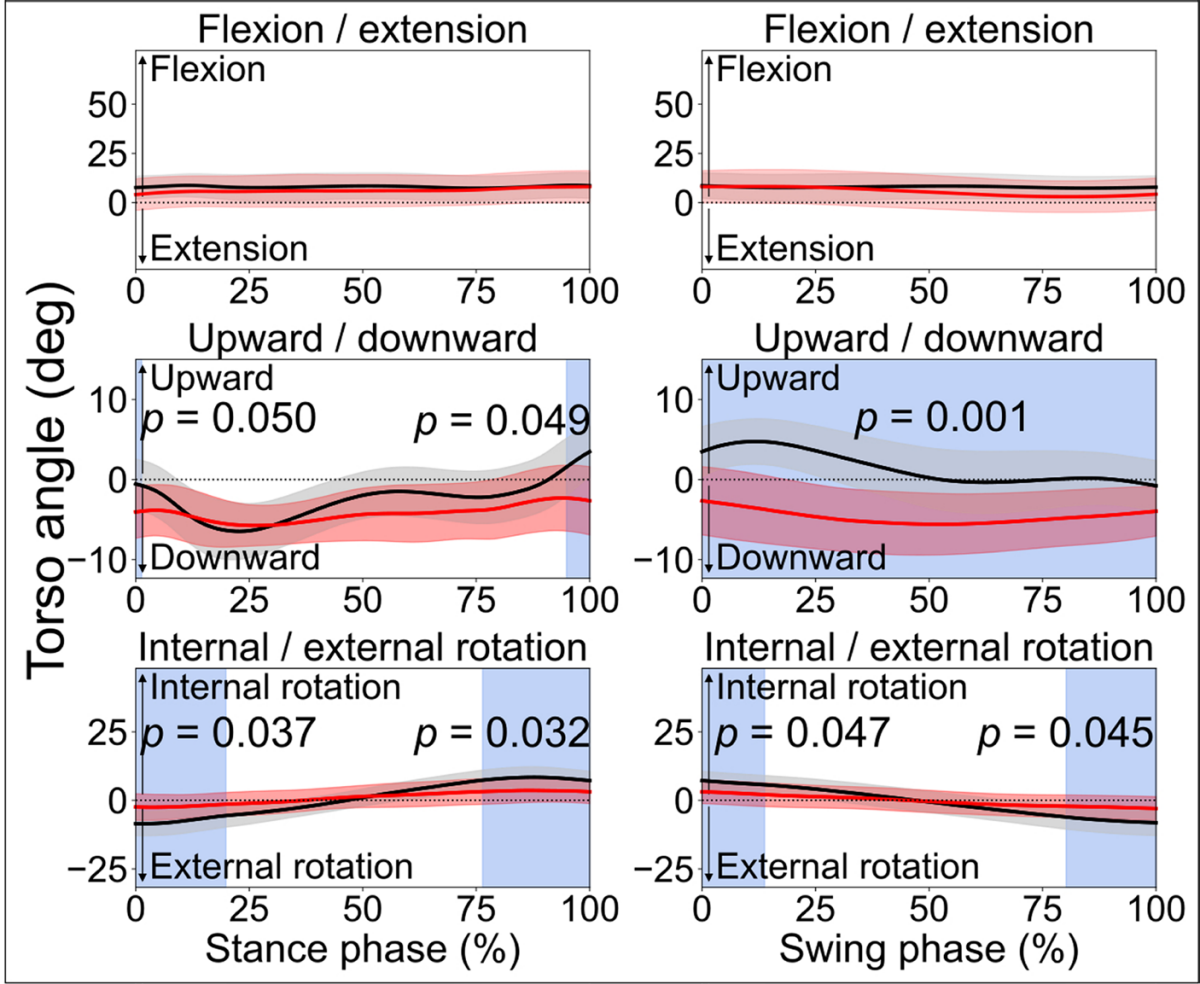
Torso angles. Comparisons between the stroke group (red lines) and control group (black lines) in the stance phase and swing phase of a gait cycle. In the frontal plane, leaning downward indicates leaning towards the affected side, while leaning upward indicates leaning toward the less-affected side. Blue shades indicate the time clusters with significant differences between the two groups (*p* < 0.05).

**Figure 3:**
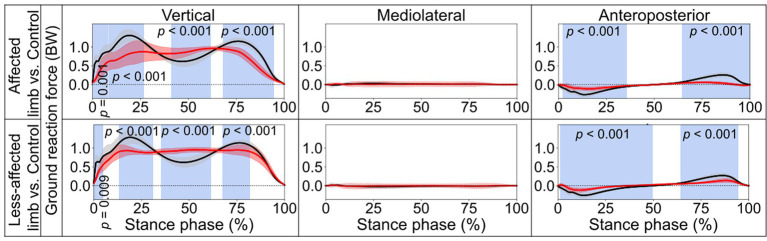
Ground reaction forces. Comparisons between the stroke group (red lines) and control group (black lines) in the stance phase of a gait cycle. Blue shades indicate the time clusters with significant differences between the two groups (*p* < 0.05). Data were normalized to the individual body weight (BW).

**Figure 4:**
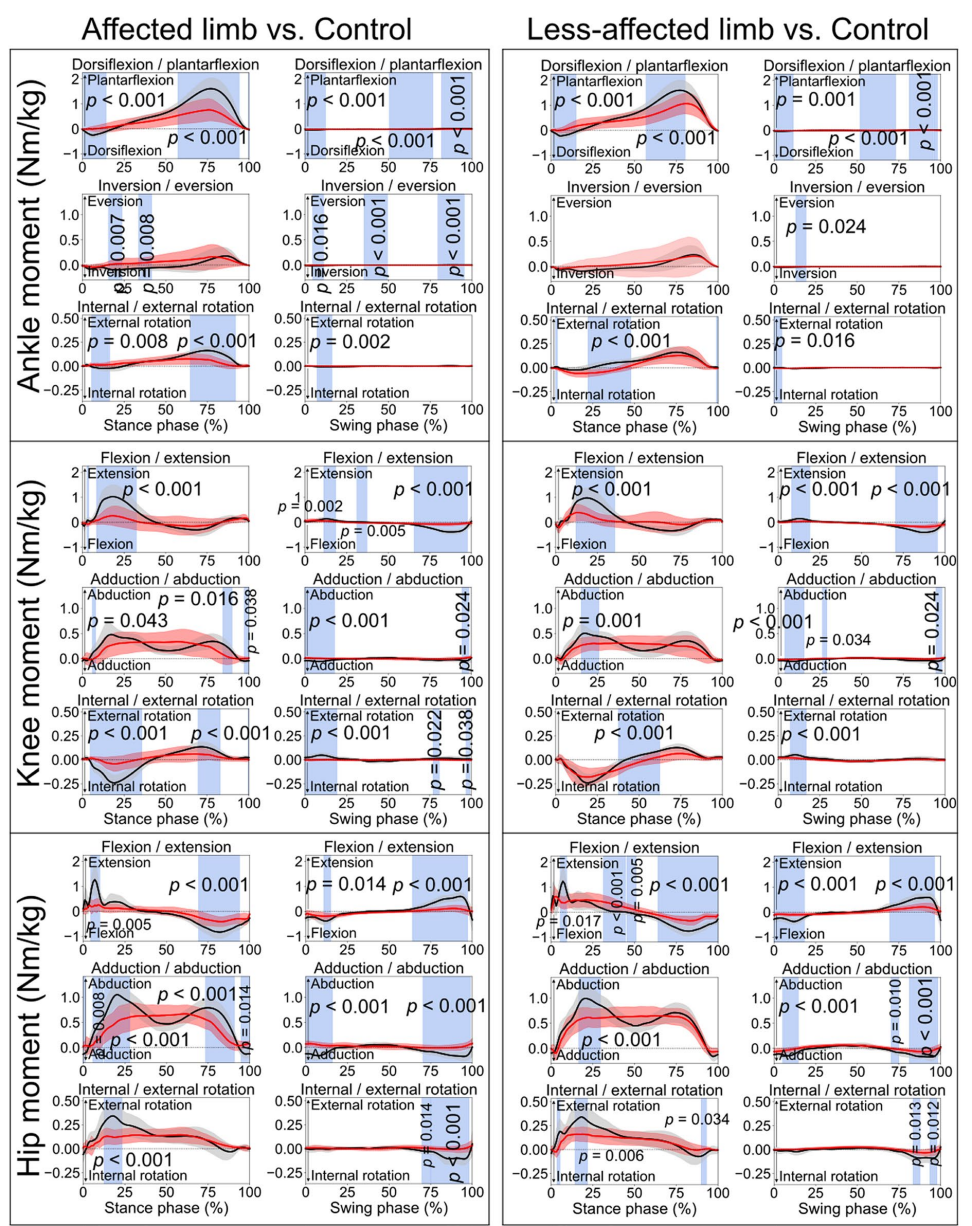
Lower limb joint moments. Comparisons between the stroke group (red lines) and control group (black lines) in the stance phase and swing phase of a gait cycle. Blue shades indicate the time clusters with significant differences between the two groups (*p <* 0.05).

All 15 stroke and 15 control participants completed the 10-m walking task. The time-varying joint angles for the lower limbs (Figure 1) and torso (Figure 2), GRF (Figure 3), and joint moments (Figure 4) are presented for both the stroke and control groups.

When comparing the joint angles and moments between the stroke patients’ affected limb and control group, SPM identified significant differences in various periods in both the stance phase and swing phase for the ankle, knee, and hip joints (all *p* < 0.05). Differences in torso angles were seen primarily not in the sagittal plane but in the frontal plane, where the torso exhibited greater downward angles in the early and terminal stance phases (*p* = 0.050 and *p* = 0.049, respectively) and entire swing phase (*p* = 0.001), indicating the stroke patients leaning towards their affected limb compared with the control group (Figure 2).

In the stance phase, significant differences in the vertical component of GRF were identified when contrasting the stroke and control groups (Figure 3). For the affected limb, the differences ranged from 0.4 to 7.8% (*p =* 0.001), from 8.3 to 26.7% (*p <* 0.001), from 41.2 to 61.6% (*p <* 0.001), and from 68.2 to 94.5% (*p <* 0.001). Similarly, for the less-affected limb, from 0.1 to 4.8% (*p =* 0.009), from 13.3 to 30.9% (*p <* 0.001), from 35.4 to 61.3% (*p <* 0.001), and from 67.6 to 81.7% (*p <* 0.001). Additionally, significant deviations from the control group in the anteroposterior component of GRF were noted. The differences were found for the affected limb to be from 2.3 to 35.5%, (*p <* 0.001) and from 64.6 to 99.9% (*p <* 0.001), while for the less-affected limb, the differences ranged from 1.3 to 49.5% (*p <* 0.001) and from 64.3 to 94.4% (*p <* 0.001).

## 4 Discussion

This study conducted a comprehensive comparison of kinematic and kinetic variables during a 10-meter walking task between a group of stroke patients and appropriately matched healthy controls. SPM was employed to assess biomechanical variables for both the stance and swing phases in the gait cycle. The findings indicated notable disparities in joint angles (Figure 1) and moments (Figure 4) during both the stance and swing phases when comparing the affected limb with the control group. Importantly, differences were also evident between the less-affected limb of the stroke group and the control participants’ matched limb. Furthermore, significant differences emerged in the amplitudes of the vertical and anteroposterior components of GRF during the stance phase (Figure 3). In contrast, no statistically significant difference was observed in the mediolateral component throughout the stance phase.

### 4.1 Joint angles

The lower limb joint angle profiles were similar to the ones reported in previous studies (Balaban and Tok, 2014; Tamaya et al., 2020). Significant differences between stroke and control participants were observed in the ankle, knee, and hip joints (Figure 1), primarily in the sagittal plane during the early stance phase, and the period from late stance to early swing phase. The former differences were likely related to weight acceptance, and the latter could be associated with foot push-off (Perry and Burnfield, 2010). During the early swing phase (0–69%, Figure 1), the affected knee displayed much smaller ROM of flexion/extension angle (< 10°) than the control group (~20°). This is consistent with stroke-related stiff knee gait or an extensor gait pattern, characterized by reduced flexion during the swing phase (Woolley, 2001; Perry and Burnfield, 2010). Thus, joint ROM recovery, in particular for the period from late stance phase to early swing phase, can be meaningful for the enhancement of walking performance (Teixeira-Salmela et al., 2001; Nesi et al., 2023).

Abnormalities were also observed in the early and late stance phases, and early swing phase of the contralateral less-affected lower limb (Figure 1). This is in line with one prior study which reported a decrease in lower limb extension angles in the late stance phase for both affected and less-affected limbs (Teixeira-Salmela et al., 2001). However, that study solely analyzed the maximum joint angles, which makes it difficult to diagnose abnormality at different period during ambulation. Hence, this reaffirms the merits of SPM in identifying differences in time-varying data (Pataky, 2010, 2012). On average, torso angles in the frontal plane demonstrated significant differences in the terminal stance phase and the entire swing phase, i.e., leaning more toward the affected side (shown as a downward angle, Figure 2), presumably as a form of compensatory strategy, soft-tissue architecture restriction, or hemi-body spasticity for the hemiplegic gait. Future studies are recommended to include electromyography (EMG) analysis to help better understand stroke patients’ torso movement deviations.

### 4.2 Ground reaction forces

The control group exhibited two obvious peaks in the vertical component of the GRF (black lines in Figure 3), which correspond to the moment of body weight transfer and the foot pushing off the ground (ankle plantarflexion). In contrast, stroke patients’ affected foot displayed significant reductions in both peaks during the stance phase, especially for the second peak, which is important for the pre-swing. This parallels the findings from the pediatric cerebral palsy research (Williams et al., 2011), which also noted reduced vertical GRF at the second peak. This also aligns patients’ reduced weight transfer to the affected foot and diminished ankle ROM during late stance compared with the controls, as shown in the present study. Interestingly, the vertical component of the GRF of the less-affected side also showed a significant reduction similar to the affected side (Figure 3). Hence, the decline of the vertical GRF of both feet may together contribute to stroke patients’ impaired dynamic gait function.

### 4.3 Joint moments

For the controls, an ankle dorsiflexion moment was generated primarily by the ankle dorsiflexor muscles (e.g., tibialis anterior) immediately after initial foot contact (early stance phase, black lines in Figure 4), serving as weight acceptance (Sloot and Van Der Krogt, 2016) when the body weight is transferred to the standing leg. However, smaller ankle dorsiflexion moments were found in both the affected limb (from 0.6 to 14.3%, *p* < 0.001) and less-affected limb (from 0.9 to 15%, *p* < 0.001) in the stroke group. This could also be reflected by the missing first vertical GRF peak in the stroke patients (Figure 3). Then, when pushing off the ground, a great plantarflexion moment was seen in the late stance phase for the control group (Sloot and Van Der Krogt, 2016). However, the affected limb of the stroke group showed smaller peak plantarflexion moments during the late stance phase (Figure 4, approximately 0.5 and 1.5 Nm/kg, respectively). This may also explain the lack of the obvious second vertical GRF peak for the stroke group (Figure 3). Clinicians, based on the current research findings, may prescribe personalized exercises, such as concentric training focusing on the ankle muscles, to improve the patients’ walking performance (Perez et al., 2024).

Similar to the ankle joint moment, a knee flexion moment (negative in value) was seen in a very short period, as shown in the early stance phase for the control group (black lines in Figure 4), which is also related to weight acceptance. Subsequently, a great extension moment (positive in value) was shown to extend the knee forward. However, consistent with the ankle joint, the stroke patients displayed smaller knee flexion and extension moments than the control group. During the stance phase, knee abduction moment is associated with stabilizing the knee joint. Hence, the stroke patients’ lower knee abduction moments than the control group (from 6 to 8%, *p* = 0.043; from 84.4 to 89.9%, *p* = 0.016; from 97.2 to 100%, *p* = 0.038) may induce an unstable knee joint during ambulation.

The controls exhibited a hip extension moment in the early stance (positive in value, black lines in Figure 4) in response to weight acceptance. After that, a hip flexion moment (negative in value) was seen in the late stance phase, which prepared the leg for push-off. However, the stroke patients’ affected limb (from 4.4 to 10%, *p* = 0.005, from 69.2 to 93.9%, *p* < 0.001) showed smaller moment amplitudes than the control group. This is consistent with the findings in a previous study that hip extension strength could be one of the most important indicators regarding stroke patients’ ability to walk independently (Smith et al., 2017). As the hip could be the leading joint while walking, stroke patients’ reduced hip extension/flexion moments may lead to weak interaction torques at the lower joints (e.g., knee and ankle) (Dounskaia, 2005). Hence, the decreased moments of the lower limb joints on both affected and less affected limbs may together contribute to the slower walking speed after stroke. This may confirm the relationship between the increases in lower limb joint moments and improved gait performance. Thus, the findings of this current study suggest that gait rehabilitation, such as joint force or muscle force recovery, should take both limbs into consideration.

Concerning the kinetic aspect in gait analysis, stroke patients exhibited diminished dorsiflexion after initial foot contact, reflected by a missing first vertical GRF peak (Figure 3), and smaller peak plantarflexion moments during late stance (Figure 4), potentially explaining the absence of the second vertical GRF peak. Additionally, stroke patients displayed reduced knee flexion and extension moments, possibly leading to unstable knee joints during ambulation. Furthermore, stroke patients exhibited lower hip extension/flexion moments, suggesting weakened interaction torques at subordinate joints and contributing to slower walking speeds in both affected and less-affected limbs. These findings emphasize the complex interplay of joint moments and GRF in stroke patients’ gait mechanics, highlighting potential targets for rehabilitation interventions aimed at improving walking function and independence.

### 4.4 Limitations

There are several limitations to this present study. Firstly, inconsistent gait patterns may be exhibited across the small sample of 15 stroke patients, and hence, future studies are recommended to recruit stroke patterns in similar status and conditions to improve group homogeneity for analysis. Secondly, since the inherent difficulty existed in obtaining valid walking trials for the stroke patients (e.g., planting the whole foot on the force platform without touching its edge), only 2 strides for each left and right foot were used for analysis. For the control group, 3 strides were included, which is in line with the previous literature (Teixeira-Salmela et al., 2001). Hence, in the future, more valid strides/trials should be included to reach a more stable mean value.

## 5 Conclusion

This study compared the kinematics (joint angles) and kinetics (GRF and joint moments) for a group of chronic stroke patients against their matched healthy controls in a 10-m walking task. SPM detected significant differences in joint angles and moments in various periods during the stance and swing phases between the stroke and controls. Between-group differences were also revealed in GRF during the stance phase. The findings reveal that in addition to the affected limb which have been extensively investigated in previous studies, the less-affected limb also exhibited abnormal biomechanics variables compared with the control group in this study. This suggests that post-stroke gait rehabilitation should take both limbs into consideration, and clinicians can prescribe personalized exercises to improve stroke patients’ walking performance. The present study illustrates that 3D motion capture technology and SPM analyses can offer clinicians valuable insights into gait pattern deviations across different phases in the gait cycle. The research findings may draw attention to specific periods within the gait cycle (e.g., early stance phase for the knee, and early swing phase for the ankle), and potentially enhances rehabilitation therapy by monitoring the responses to therapeutic modalities.

## Data availability statement

The raw data supporting the conclusions of this article will be made available by the authors, without undue reservation.

## Ethics statement

The studies involving humans were approved by National Healthcare Group Domain Specific Review Board, Singapore. The studies were conducted in accordance with the local legislation and institutional requirements. The participants provided their written informed consent to participate in this study.

## Author contributions

JP: Data curation, Formal analysis, Visualization, Writing – original draft, Writing – review & editing. AS: Methodology, Supervision, Writing – original draft, Writing – review & editing. T-LW: Formal analysis, Methodology, Visualization, Writing – review & editing. WK: Conceptualization, Resources, Writing – review & editing, Investigation. PO: Conceptualization, Writing – review & editing. MT: Conceptualization, Writing – review & editing. MP: Conceptualization, Writing – review & editing. WC: Data curation, Investigation, Writing – review & editing. WA: Conceptualization, Supervision, Writing – review & editing. KC: Conceptualization, Funding acquisition, Resources, Writing – review & editing, Supervision.
