# Supplementary material for: Unraveling stroke gait deviations with movement analytics, more than meets the eye: a case control study
Source: Front Neurosci. 2024 Jul 22;18:1425183. doi: 10.3389/fnins.2024.1425183 (PMC11298395; doi:10.3389/fnins.2024.1425183)
Supplement: Supplementary file 1 [file Table_1.DOCX]

Supplementary Material


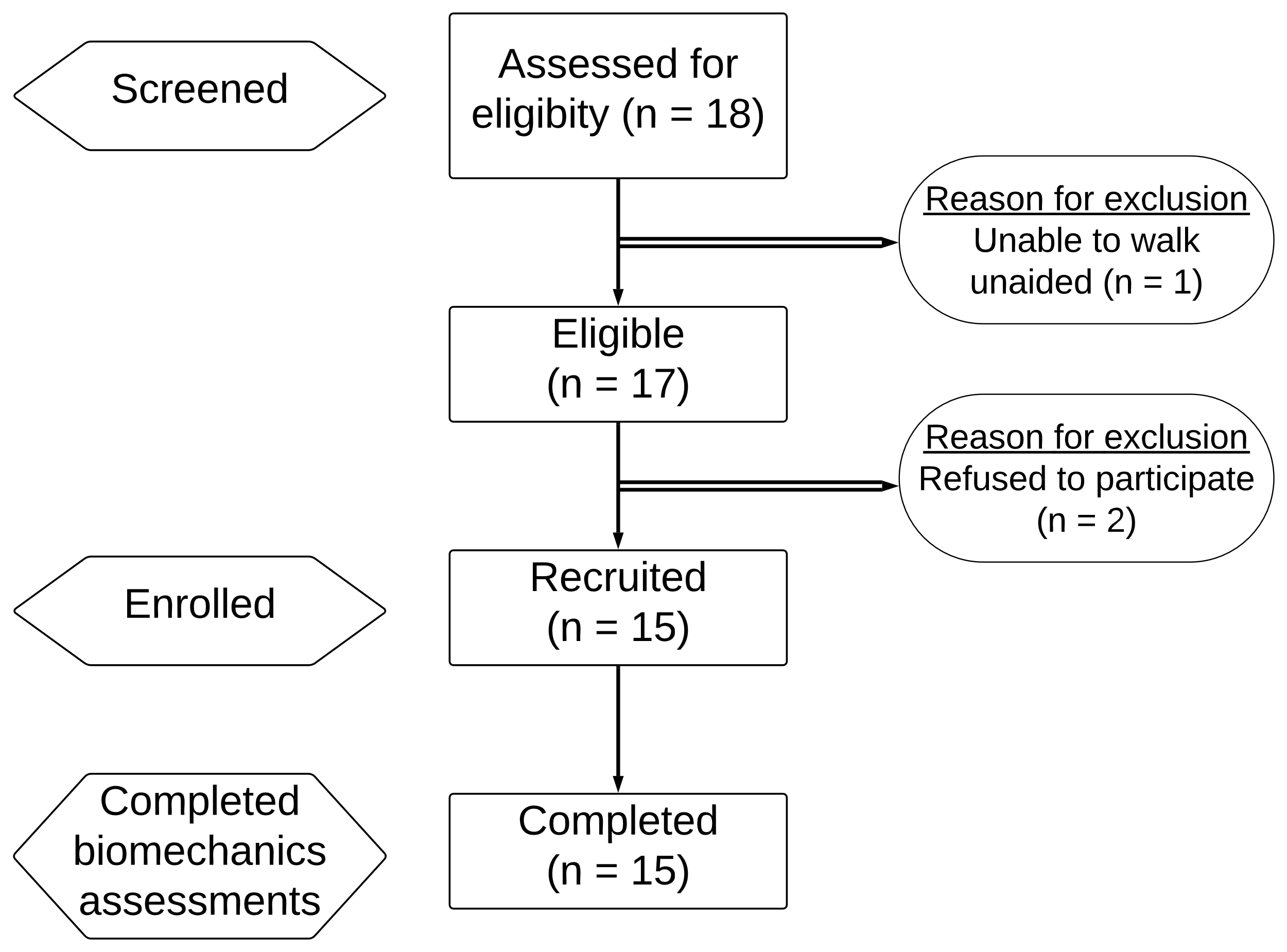


**Figure S1.** Stroke patient recruitment.

**Inclusion criteria for the stroke group**

All stroke participants were screened for study eligibility by clinicians according to the following the inclusion criteria: (1) first unilateral stroke diagnosed by neurologists or neurosurgeons and confirmed with brain imaging [computed tomography (CT) or magnetic resonance imaging (MRI)], (2) stroke duration > 6 months, (3) ambulant with at most contact guard/standby supervision without walking aid prior to the study, (4) minimum walking speed ≥ 0.2 m/s obtained on the 10-m walking test, (5) able to walk ≥ 50 m on the 6-min walk test, (6) ≥ 4 for the Functional Ambulation Category, (7) cognitive ability scored > 6/10 for the Abbreviated Mental Test, (8) able to understand the study procedures and sign informed consent, and (9) able to attend a single 2.5-h session of research data collection.

Stroke participants were excluded if they had any of the following conditions: unstable or recent cardiorespiratory conditions including uncontrolled hypertension/hypotension, angina pectoris, myocardial infarction, active congestive cardiac failure, untreated cardiac arrhythmias (e.g., atrial fibrillation), untreated pulmonary embolism or deep vein thrombosis, or presence of an implanted cardiac pacemaker. In addition, the following conditions were also excluded: local limb conditions which could be exacerbated by research conditions (e.g., walking tasks and application of adhesive skin markers); open wounds, skin ulcers, uncontrolled eczema, psoriasis, fungal, or bacterial infections, active arthritis or joint or limb pain, presence of severe aphasia or neglect, cognitive impairment, dementia, untreated depression, psychiatric disorder, end stage conditions such as medical instability or orthostatic insufficiency, organ, renal, liver, heart failure, hemodialysis and life expectancy less than 6 months. Pregnant and lactating females were also excluded.

**Table S1.** Individual demographic and clinical characteristics for the stroke patients (n = 15).

| No. | Age (years) | Sex | Ethnicity | Stand height (cm) | Body mass (kg) | Stroke diagnosis | Side | Speed (m/s) | D-6  (m) | FMA | AFO |
| --- | --- | --- | --- | --- | --- | --- | --- | --- | --- | --- | --- |
| 1 | 50 | M | Myanmarese | 181 | 88.4 | Hemorrhage | R | 0.45 | 149 | 28 | N |
| 2 | 42 | M | Chinese | 170 | 61.8 | Infarct | R | 1.13 | 344 | 33 | N |
| 3 | 67 | M | Indian | 177 | 73.7 | Hemorrhage | L | 0.94 | 259 | 31 | N |
| 4 | 58 | M | Chinese | 183 | 63.8 | Infarct | L | 1.00 | 267 | 21 | Y |
| 5 | 63 | M | Chinese | 172 | 69.4 | Hemorrhage | L | 1.20 | 330 | 27 | N |
| 6 | 51 | M | Chinese | 172 | 70 | Infarct | R | 1.00 | 270 | 33 | N |
| 7 | 55 | M | Chinese | 172 | 67.9 | Hemorrhage | R | 1.04 | 300 | 27 | N |
| 8 | 38 | F | Nepalese | 162 | 50.9 | Hemorrhage | L | 1.00 | 356 | 28 | Y |
| 9 | 61 | M | Chinese | 174 | 72.4 | Infarct | L | 1.00 | 248 | 30 | N |
| 10 | 29 | F | Chinese | 159 | 64.3 | Hemorrhage | R | 1.16 | 357 | 23 | Y |
| 11 | 56 | F | Chinese | 154 | 56.8 | Infarct | R | 1.00 | 220 | 33 | N |
| 12 | 64 | M | Chinese | 168 | 71.3 | Infarct | R | 0.54 | 160 | 26 | N |
| 13 | 58 | M | Chinese | 170 | 67 | Hemorrhage | R | 0.75 | 217 | 26 | N |
| 14 | 40 | M | Chinese | 166 | 58.3 | Infarct | R | 0.75 | 255 | 23 | Y |
| 15 | 74 | F | Chinese | 148 | 44.3 | Infarct | R | 0.55 | 180 | 30 | N |

Note. F denotes female; M denotes male; Y denotes yes; N denotes no; R denotes right; L denotes left. Side denotes the body side affected (paretic side). Speed denotes the mean speed in the 10-m walking task. D-6 denotes the total walking distance in the 6-min walking distance. FMA denotes Fugl-Meyer Assessment. AFO denotes whether the stroke patient wore an ankle foot orthosis or not during the experiment sessions.
